# Work alienation among young university teachers in China

**DOI:** 10.3389/fpsyg.2026.1845561

**Published:** 2026-08-28

**Authors:** Jing Li, Danlei Lin, Tingting Chang

**Affiliations:** School of Education, Guangzhou University, Guangzhou, China

**Keywords:** Chinese higher education, early-career academics, job characteristics, perceived organizational environment, work alienation, young university teachers

## Abstract

**Introduction:**

Young university teachers in China often begin their careers under competitive employment arrangements that combine intensive teaching and research demands with uncertain prospects for long-term advancement.

**Methods:**

Using cross-sectional survey data from 532 young university teachers in Chinese undergraduate institutions, this study examined how adverse job characteristics, background characteristics, and perceived organizational environment were associated with work alienation. Confirmatory factor analysis, measurement validation, integrated regression models, and moderation analyses were conducted.

**Results:**

After institutional clustering was considered, lack of support and fairness, perceived busyness, and self-bias/perceived prejudice remained positively associated with work alienation, whereas the association for interpersonal strain and conflict was not robust. Perceived organizational environment was negatively associated with work alienation, but the proposed moderation was not supported.

**Discussion:**

The findings identify associations between perceived work conditions, perceived organizational environment, and work alienation. Given the cross-sectional design, causal and temporal interpretations require longitudinal or experimental evidence.

## 1 Introduction

Young university teachers in China provide a timely and theoretically meaningful context for examining work alienation in contemporary higher education. In this study, young university teachers refer to early-career faculty members aged 40 or below, consistent with the sampling criteria used in the present survey. According to the Ministry of Education’s 2024 national statistical bulletin on education development, China had 2.1635 million full-time teachers in higher education, including 1.3876 million in regular undergraduate institutions (Ministry of Education, 2025). Within this large academic workforce, young teachers are expected to contribute to teaching, research, student development, and institutional renewal. Yet their early academic careers are increasingly shaped by competitive employment arrangements, performance-based evaluation, and uncertain advancement prospects. In Chinese universities, the transition from more stable “steel bowl” employment to contract-based “up-or-out” systems has been associated with intensified accountability, quantified productivity expectations, and high-stakes career assessment (Si, 2023; Yin and Mu, 2023). Although these reforms may be intended to improve institutional competitiveness and academic productivity, they also create conditions in which early-career academics must pursue professional development under strong uncertainty and pressure.

This issue has also become visible in recent policy discussions. The Guiding Opinions on Strengthening the Development of Young University Teachers in the New Era, issued by the Ministry of Education and five other departments, call for improving appointment mechanisms, scientifically setting pre-tenure periods, strengthening process management and feedback, increasing support for young faculty members, reforming evaluation systems, and reducing non-teaching and non-research burdens (Ministry of Education et al., 2025). These policy emphases suggest that young teachers’ work experiences are not merely matters of individual adaptation. Rather, they are closely connected to how academic work is organized, evaluated, and supported within higher education institutions. Recent research on early-career academics in China similarly shows that organizational support, including instrumental, emotional, and academic support, is important for their development, although academic support is often insufficient for sustained professional growth (Wang et al., 2025). Therefore, young university teachers in China constitute an important group for studying how work organization and organizational experience are associated with alienation.

Work alienation offers a useful conceptual lens for this purpose. Classical alienation theory identifies powerlessness, meaninglessness, normlessness, social isolation, and self-estrangement as central dimensions of alienation (Seeman, 1959; Dean, 1961). Later organizational research understands work alienation as a state in which employees become detached from work because it no longer fulfils their needs, values, or expectations (Banai and Reisel, 2007). In academic work, this concept is especially relevant because teaching and research are closely tied to professional identity, intellectual autonomy, social contribution, and a sense of vocation. Therefore, when teachers experience their work as externally controlled, weakly recognized, socially isolating, or disconnected from professional purpose, the issue is not limited to stress, burnout, or job dissatisfaction. It may involve a deeper rupture between teachers and the academic work through which they construct professional value.

General work-design theories provide an important starting point for understanding this rupture. Classical work-design research suggests that task significance, autonomy, and feedback shape motivation and psychological attachment to work (Hackman and Oldham, 1976), while later research highlights the role of social and contextual work characteristics in well-being and engagement (Humphrey et al., 2007). The job demands–resources perspective similarly argues that demanding work conditions may undermine employees’ psychological connection to work, whereas adequate resources can help buffer these effects (Demerouti et al., 2001; Bakker and Demerouti, 2007). However, although these theories identify important mechanisms, they do not fully explain how such mechanisms operate when work is simultaneously professional, identity-laden, and governed by institutional evaluation.

Academic work therefore requires contextual re-examination. Unlike many occupational groups, university teachers are evaluated not only as employees but also as intellectual and professional actors whose work is judged through teaching quality, research productivity, collegial recognition, and institutional contribution. Autonomy in this context is not simply a motivational resource; it is also a normative foundation of academic professionalism. Similarly, collegial relations and organizational recognition matter not only because they make work more pleasant, but also because they shape access to mentoring, networks, resources, and academic belonging. Institutional evaluation is also especially consequential, since it directly affects promotion, tenure prospects, research opportunities, and long-term career security. Thus, adverse job characteristics in universities may contribute to alienation not only by increasing strain, but also by weakening teachers’ sense of professional autonomy, recognition, and belonging.

Recent studies on academic work support this context-sensitive view. Watermeyer et al. (2024) show that deteriorating working conditions, weak leadership, and the erosion of meaning may contribute to academic anomie in post-COVID higher education. Bougherza et al. (2024) demonstrate that the internal work environment is closely associated with faculty members’ job alienation. Marques et al. (2024) show that higher education change reshapes academic identities, while Naidoo-Chetty and du Plessis (2021) highlight the distinctive configuration of job demands and job resources experienced by academics. Studies of Chinese higher education further indicate that tenure-track and “up-or-out” arrangements may generate tensions around resource competition, career uncertainty, workload intensification, and identity construction among young academics (Si, 2023; Yin and Mu, 2023). Together, these studies suggest that academic work should not be treated simply as another occupational setting; its professional, relational, and institutional features may shape the formation of work alienation in specific ways. More specifically, the present study engages with these studies as points of comparison rather than as stand-alone citations. It confirms Bougherza et al.’s (2024) emphasis on the importance of the internal work environment, extends Naidoo-Chetty and du Plessis’s (2021) job demands–resources perspective by examining the alienating consequences of multiple adverse job characteristics, and connects Marques et al.’s (2024) academic identity perspective to alienation by showing how misrecognition, weak belonging, and limited autonomy may weaken teachers’ professional identification. At the same time, it differs from Watermeyer et al. (2024), Si (2023), and Yin and Mu (2023) by focusing not on academic anomie, tenure-track adaptation, or resilience, but on work alienation as a measurable outcome among young university teachers in China.

Despite these insights, three gaps remain. First, although work-design and job demands–resources research has explained how job characteristics influence employees’ psychological connection to work, less is known about how these mechanisms operate in academic work, where professional identity, autonomy, collegial recognition, and institutional evaluation are especially salient. Second, studies of academic work and academic identity have documented the pressures facing academics, but they have not always examined work alienation as a distinct outcome involving meaninglessness, powerlessness, normlessness, social alienation, and self-estrangement. Third, previous research has often examined job-related pressures, individual background, or organizational environment separately, leaving insufficient understanding of how these domains jointly shape alienation among young university teachers in China. Accordingly, the research gap is not simply the absence of studies on Chinese young academics. Rather, the gap lies in the limited integration of several related literatures. Prior research has examined academic anomie, faculty job alienation, job demands and resources, academic identity, and organizational support, but these strands have rarely been brought together to explain how adverse job characteristics and perceived organizational environment jointly shape work alienation. The present study therefore treats prior literature as a foundation for specifying what is confirmed, extended, and differentiated in this context.

To address these gaps, the present study examines work alienation among young university teachers in Chinese undergraduate institutions. It focuses on three interrelated domains: adverse job characteristics, teachers’ background characteristics, and perceived organizational environment. Adverse job characteristics capture negative aspects of academic work experience, including lack of support and fairness, perceived busyness, interpersonal strain and conflict, and self-bias or perceived prejudice. Background characteristics reflect teachers’ social and professional positions, such as gender, age, marital status, rank, institutional type, discipline, and income. Perceived organizational environment captures teachers’ evaluation of whether the workplace is supportive, harmonious, affirming, and opportunity-enhancing. By bringing these domains together, the study seeks to provide a more integrated explanation of work alienation among young university teachers.

Specifically, this study addresses the following research questions:

*RQ1*: Which dimensions of adverse job characteristics are most strongly associated with work alienation among young university teachers?

*RQ2*: To what extent are teachers’ background characteristics associated with variation in work alienation?

*RQ3*: How is perceived organizational environment associated with work alienation, and does it moderate the association between adverse job characteristics and work alienation?

This study contributes to the literature in four ways. First, it provides context-specific evidence on young university teachers in China, a group that is institutionally important but remains insufficiently examined in work alienation research. Second, it extends work alienation theory to the academic workplace by showing how alienation may be connected to the organization, evaluation, and recognition of academic work. Third, it integrates adverse job characteristics, background characteristics, and perceived organizational environment within one analytical framework, thereby moving beyond single-factor explanations of alienation. Fourth, it examines perceived organizational environment not only as a direct organizational condition but also as a potential buffer that may weaken the association between adverse job characteristics and work alienation. More specifically, the study confirms previous research showing that internal work environment and organizational support are closely related to alienation and work well-being (Bougherza et al., 2024; Wang et al., 2025). It extends the job demands-resources literature (Naidoo-Chetty and du Plessis, 2021) by evaluating which adverse job-characteristic dimensions remain associated with alienation when considered simultaneously and after institutional clustering is taken into account. It also extends academic identity research by linking identity-related experiences of recognition, belonging, and professional meaning to work alienation. Finally, it differs from prior research on academic anomie and Chinese tenure-track experiences (Watermeyer et al., 2024; Si, 2023; Yin and Mu, 2023) by providing an integrated quantitative model of alienation among young university teachers in China.

The remainder of the article is organized as follows. The next section reviews the theoretical literature on adverse job characteristics, background characteristics, perceived organizational environment, and work alienation, and develops the hypotheses. The methods section describes the sample, measures, and analytical procedures. The results section reports the measurement models, structural relationships, background-variable analyses, and moderation test. The discussion section interprets the findings in relation to prior research and considers implications, limitations, and directions for future research.

## 2 Theory and hypotheses

The present study conceptualizes work alienation among young university teachers as a relational outcome shaped by work organization, individual position, and perceived organizational environment. This framework builds on three complementary theoretical perspectives. First, work-design theory suggests that employees’ psychological attachment to work is shaped by the structure and quality of their job characteristics, including autonomy, task significance, feedback, and social-contextual work features (Hackman and Oldham, 1976; Humphrey et al., 2007). Second, the job demands–resources perspective argues that demanding work conditions can undermine well-being and engagement, whereas social and organizational resources can reduce the harmful effects of such demands (Demerouti et al., 2001; Bakker and Demerouti, 2007). Third, social information processing theory suggests that employees interpret their work through cues from the organizational environment, including fairness, support, recognition, and relational climate (Salancik and Pfeffer, 1978).

These perspectives are particularly useful for examining work alienation in higher education because academic work is not only task-based but also identity-laden, relational, and institutionally evaluated. For university teachers, work is closely connected with professional identity, academic autonomy, collegial recognition, and long-term career development. Therefore, unfavorable work conditions may not only create stress or dissatisfaction but also weaken teachers’ sense of meaning, control, belonging, and identification with academic work. The following sections develop hypotheses concerning adverse job characteristics and perceived organizational environment, while also explaining why teachers’ background characteristics are treated as an exploratory domain.

### 2.1 Adverse job characteristics and work alienation

Job characteristics constitute the most immediate level at which university teachers experience institutional expectations in everyday academic work. In work-design research, job characteristics are not limited to isolated task attributes; rather, they include motivational, social, and contextual conditions that shape how individuals experience work as meaningful, manageable, supported, and self-directed (Hackman and Oldham, 1976; Humphrey et al., 2007). From a job demands–resources perspective, adverse job conditions are likely to erode psychological attachment to work when they increase demands without providing corresponding resources, support, or recognition (Demerouti et al., 2001; Bakker and Demerouti, 2007). In this sense, work alienation may emerge when the organization of academic work repeatedly weakens teachers’ sense of autonomy, fairness, relational security, and professional meaning.

In the present study, adverse job characteristics include four dimensions: lack of support and fairness, perceived busyness, interpersonal strain and conflict, and self-bias or perceived prejudice, the latter referring to negative self-perception and perceived misrecognition within the institution. These dimensions capture different but related ways in which academic work may become alienating. Lack of support and fairness reflects teachers’ perceptions that leadership support, institutional recognition, resource allocation, and evaluation procedures are inadequate or unfair. Organizational justice research shows that fairness perceptions are strongly associated with employees’ attitudes, strain, commitment, and withdrawal reactions (Colquitt et al., 2001; Cohen-Charash and Spector, 2001). Cole et al. (2010) further showed that distributive and interpersonal justice are related to emotional exhaustion and withdrawal outcomes. In educational settings, Ji et al. (2025) similarly found that teachers’ perceived organizational justice is associated with lower burnout and more positive organizational behavior. These findings suggest that when young university teachers perceive a lack of support or fairness, they may experience academic work as less legitimate, less valued, and less meaningful.

Interpersonal strain and conflict represent another important pathway to alienation. Academic work is deeply relational: university teachers interact with colleagues, students, supervisors, administrators, and external evaluators. These relationships can provide mentoring, recognition, and belonging, but they can also become sources of conflict, emotional depletion, and social isolation. Prior research has shown that organizational injustice and strained interpersonal relations can contribute to work alienation and reduced organizational commitment (Sulu et al., 2010). In higher education, Bougherza et al. (2024) also found that the internal work environment is closely associated with faculty members’ job alienation. For young academics, interpersonal conflict may be particularly consequential because collegial relations and mentoring networks often shape access to resources, academic opportunities, and professional development.

Perceived busyness reflects the chronic intensification and fragmentation of academic work. Young university teachers are often expected to balance teaching, research, student supervision, administrative duties, service work, and performance evaluation within compressed time frames. Studies on academics’ job demands and resources show that workload, emotional demands, administrative pressure, and limited resources are central features of academic work and may undermine well-being when not adequately supported (Naidoo-Chetty and du Plessis, 2021). Recent studies of early- and mid-career academics also show that excessive workload, limited support, and unclear career pathways are major challenges for academic flourishing and work well-being (Stratford et al., 2024; Piano et al., 2024). In the Chinese higher education context, the “up-or-out” system may further intensify time pressure because early-career academics must accumulate measurable outputs within a limited period (Yin and Mu, 2023). Under such conditions, busyness is not merely a matter of having many tasks; it may reduce teachers’ sense that academic work is self-directed, coherent, and intellectually meaningful.

Self-bias and perceived prejudice concern teachers’ perceptions that they are not fully recognized, fairly evaluated, or treated as legitimate members of the institution. These experiences may arise from age, gender, rank, disciplinary status, institutional position, or perceived mismatch between personal aspirations and organizational expectations. Research on academic identity suggests that higher education change reshapes how academics understand their professional roles and values (Marques et al., 2024). Recent research on higher education teachers also shows that professional identity is shaped through ongoing negotiation between personal commitment, institutional conditions, recognition, leadership, and the value assigned to teaching work (Halal Orfali et al., 2024). Therefore, when young teachers experience misrecognition, perceived prejudice, or internalized self-doubt, they may become alienated not only from the organization but also from their own sense of professional worth.

Taken together, these four dimensions indicate that adverse job characteristics may contribute to work alienation through multiple mechanisms: weakening perceived fairness, increasing chronic strain, undermining relational belonging, and destabilizing professional identity. Therefore, the first hypothesis is proposed:

*H1*. Adverse job characteristics are positively associated with work alienation among young university teachers. More specifically, lack of support and fairness, perceived busyness, interpersonal strain and conflict, and self-bias/perceived prejudice are expected to show positive associations with work alienation.

### 2.2 Teachers’ background characteristics and variation in work alienation

Although job characteristics are central to understanding work alienation, teachers do not encounter academic work from identical social or professional positions. Background characteristics may shape teachers’ access to support, exposure to institutional vulnerability, interpretation of evaluation pressure, and ability to mobilize coping resources. Therefore, teachers’ background characteristics should not be treated only as residual demographic controls; they may help explain why alienation varies across groups.

Several background characteristics are relevant in this regard. Gender may shape academic experience through service expectations, informal requests, recognition, and the distribution of academic labor (O’Meara et al., 2019), although its effects should not be interpreted in essentialist terms. Age and career stage are also important because early-career academics often have less accumulated institutional capital, weaker professional networks, and greater uncertainty about future advancement. Studies of early-career academics show that flourishing and work well-being are closely tied to workload, mentoring, career development opportunities, and people-centered organizational cultures (Stratford et al., 2024; Piano et al., 2024). In Chinese higher education, young academics working under up-or-out arrangements may also face high-stakes evaluation and pressure to accumulate academic capital within a short period (Yin and Mu, 2023).

Institutional and socioeconomic positions may further shape alienation. Institutional level, academic rank, discipline, administrative role, marital status, and income may influence teachers’ access to research funding, graduate students, mentoring networks, promotion opportunities, material security, and social support. However, the effects of these characteristics are likely to be complex and context-dependent. They may indicate teachers’ different locations within academic and social hierarchies, but they do not necessarily imply uniform or deterministic effects on work alienation.

For this reason, the present study does not develop a directional hypothesis for each background characteristic. Rather, it treats this domain as exploratory and examines whether work alienation is patterned by teachers’ social and professional positions. This approach avoids assuming that demographic variables operate uniformly while still recognizing that background characteristics may shape vulnerability to alienation. Accordingly, the study asks:

*RQ2*. To what extent are teachers’ background characteristics associated with variation in work alienation?

### 2.3 Perceived organizational environment and work alienation

Perceived organizational environment refers to teachers’ overall evaluation of whether their workplace is supportive, harmonious, affirming, and opportunity-enhancing. Unlike formal organizational structures alone, perceived organizational environment captures how teachers interpret everyday organizational life, including interpersonal climate, recognition, communication, support, and developmental opportunities. Social information processing theory suggests that employees form attitudes and behavioral orientations partly through cues provided by their work environment (Salancik and Pfeffer, 1978). Thus, teachers’ perceptions of the organizational environment may shape whether institutional arrangements are experienced as enabling, fair, and meaningful or as constraining, indifferent, and alienating.

A positive organizational environment can reduce work alienation for several reasons. First, supportive environments provide socio-emotional resources that help teachers cope with demands and maintain a sense of professional value. Perceived organizational support theory suggests that employees’ beliefs about whether the organization values their contributions and cares about their well-being are associated with commitment, diligence, and positive work attitudes (Eisenberger et al., 1986; Eisenberger et al., 1990; Rhoades and Eisenberger, 2002). Second, a fair and supportive environment may reduce the emotional cost of work. Organizational justice research has shown that unfairness is associated with psychological strain and withdrawal reactions, whereas fairness can support healthier organizational attachment (Colquitt et al., 2001; Cole et al., 2010). Third, in academic workplaces, organizational environment is closely tied to belonging, recognition, and academic identity. When teachers feel respected, supported, and provided with meaningful opportunities, they are more likely to experience academic work as connected to professional development and self-realization.

Recent empirical evidence supports this reasoning in educational and higher education contexts. Korumaz et al. (2020) found that school culture predicts teachers’ alienation, suggesting that alienation is shaped by the broader cultural and relational environment of the institution. Bougherza et al. (2024) similarly showed that the internal work environment is associated with faculty members’ job alienation. Ji et al. (2025) found that teachers’ perceived organizational justice is related to lower burnout and more positive organizational behavior. In Chinese higher education, recent evidence also indicates that early-career academics’ development depends on instrumental, emotional, and academic support, although academic support may remain insufficient for sustained growth (Wang et al., 2025). These findings suggest that perceived organizational environment is not merely a background condition; rather, it is a substantive organizational factor that can shape teachers’ psychological connection to work.

Therefore, when young university teachers perceive the organizational environment as positive, they may be less likely to feel powerless, meaningless, socially isolated, or estranged from their academic roles. Conversely, when the environment is perceived as unsupportive, conflictual, or lacking in recognition, alienation is more likely to occur. Based on this analysis, the following hypothesis is proposed:

*H2*. A more positive perceived organizational environment is negatively associated with work alienation among young university teachers.

### 2.4 The moderating role of perceived organizational environment

Beyond its direct association with work alienation, perceived organizational environment may also function as a boundary condition that shapes how adverse job characteristics are translated into alienation. The job demands–resources perspective suggests that resources are especially important when demands are high because they can buffer the negative effects of demanding work conditions on psychological states and work engagement (Bakker et al., 2007; Bakker and Demerouti, 2007). In the present study, perceived organizational environment can be understood as an organizational resource that provides support, recognition, fairness, and a sense of belonging. These resources may reduce the alienating consequences of adverse job characteristics.

A positive perceived organizational environment may weaken the relationship between adverse job characteristics and alienation in several ways. First, it can change how teachers interpret negative work experiences. According to social information processing theory, employees do not respond to work conditions mechanically; instead, they interpret these conditions through environmental cues (Salancik and Pfeffer, 1978). When the workplace climate is supportive and affirming, young teachers may interpret workload, conflict, or evaluation pressure as manageable challenges within a developmental environment rather than as signs of neglect or institutional disregard. Second, a positive environment may provide compensatory resources. Even when teachers face busyness, interpersonal strain, or perceived unfairness, access to mentoring, recognition, collegial support, and academic opportunities may help preserve their sense of belonging and professional purpose. Third, organizational environment may protect academic identity. Because academic work is closely tied to autonomy, recognition, and professional self-understanding, a supportive environment may help teachers maintain identification with academic work even when particular job characteristics are unfavorable.

By contrast, when perceived organizational environment is poor, the alienating potential of adverse job characteristics may be amplified. Lack of support and fairness may be interpreted as institutional indifference; busyness may be experienced as exploitation rather than professional development; interpersonal conflict may deepen social isolation; and perceived bias may intensify self-doubt and misrecognition. In such contexts, negative job characteristics are more likely to produce alienation because they occur within an environment that offers few countervailing resources.

This moderating logic is consistent with prior research showing that organizational resources can buffer the harmful effects of demanding conditions (Bakker et al., 2007; Bakker and Demerouti, 2007). It is also consistent with recent research on academic work showing that early-career academics’ well-being depends not only on workload but also on mentoring, career development, support, and people-centered organizational cultures (Stratford et al., 2024; Piano et al., 2024; Wang et al., 2025). Therefore, the present study conceptualizes perceived organizational environment as both a direct protective factor and a contextual resource that may weaken the association between adverse job characteristics and work alienation. Based on this reasoning, the following hypothesis is proposed:

*H3*. Perceived organizational environment moderates the positive association between adverse job characteristics and work alienation, such that the association is weaker when perceived organizational environment is more positive.

### 2.5 Conceptual model

**Figure 1:**
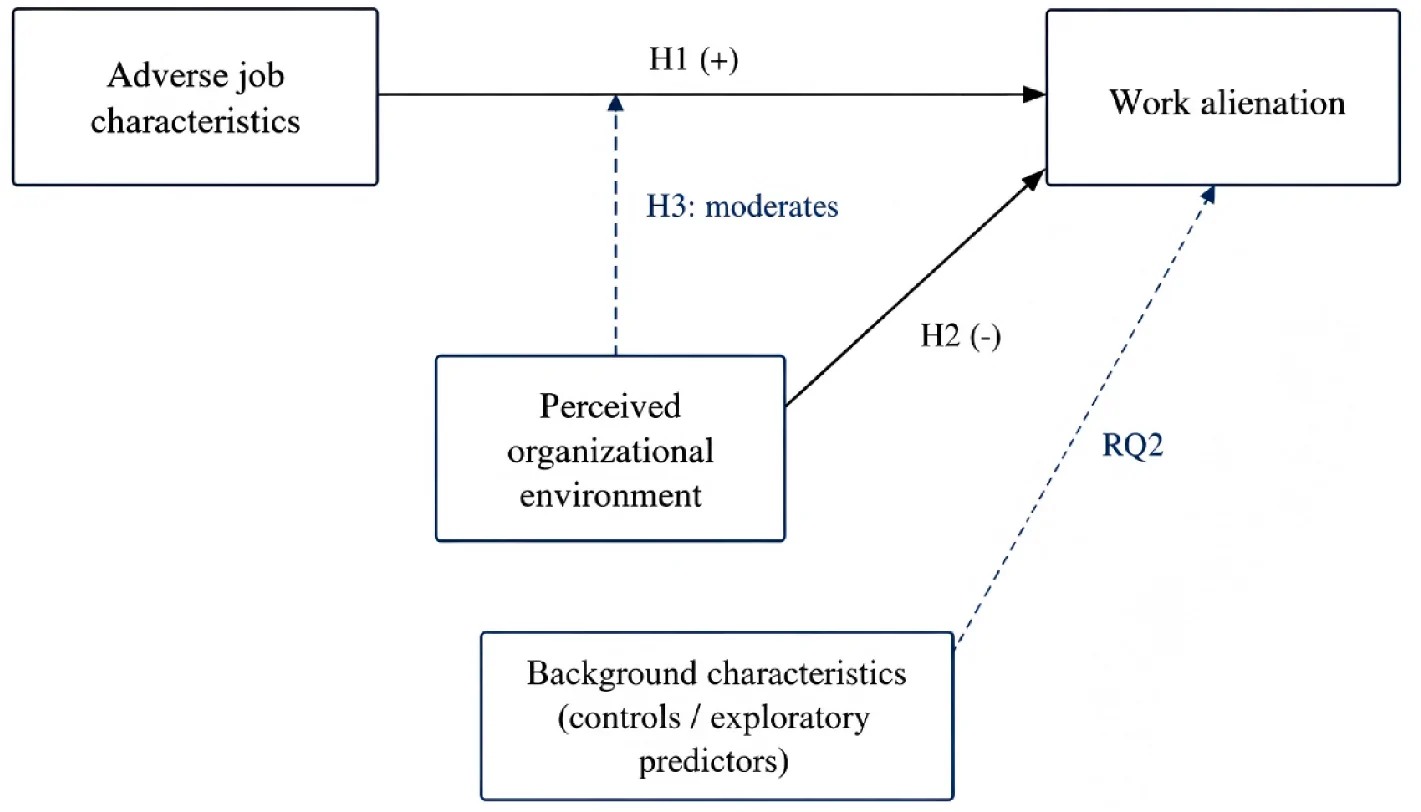
Conceptual model of work alienation among young university teachers in China. Background characteristics include gender, age, marital status, rank, institutional type, discipline, and income.

Taken together, the proposed framework suggests that work alienation among young university teachers is shaped by adverse job characteristics, teachers’ social and professional positions, and perceived organizational environment. Adverse job characteristics are expected to be positively associated with work alienation, while a more positive perceived organizational environment is expected to be negatively associated with work alienation. Perceived organizational environment is also expected to buffer the positive association between adverse job characteristics and work alienation. Teachers’ background characteristics are examined as an exploratory domain to determine whether alienation varies across social and professional positions. The conceptual model is presented in Figure 1.

## 3 Materials and methods

### 3.1 Participants

The final sample comprised 532 full-time university teachers aged 40 years or younger from eight undergraduate institutions (four in eastern, three in central, and one in western China; three Double First-Class and five non-Double First-Class). Universities were purposively selected to increase geographic and institutional variation; within institutions, anonymous invitations were distributed through human resources or research administration offices, and individual participation therefore followed convenience sampling. Of 800 invited teachers, 562 responded (70.3%); 30 questionnaires were excluded for more than 10% missing data (*n* = 22) or completion in less than 3 min (*n* = 8), leaving 532 usable cases (66.5% of those invited). Eligibility required full-time faculty status, age ≤40 years, employment at an undergraduate institution, and teaching and/or research responsibilities. Participation was voluntary and anonymous. Because neither institutions nor individuals were probability sampled, the sample was diverse but not nationally representative.

Among the valid samples, 51.5% were male teachers (*n* = 274) and 48.5% were female teachers (*n* = 258). In terms of age distribution, 27.4% were aged 30 years or younger (*n* = 146), 54.3% were aged 31–35 years (*n* = 289), and 18.2% were aged 36–40 years (*n* = 97). With respect to administrative positions, 24.8% held administrative roles (*n* = 132), whereas 75.2% did not (*n* = 400). Participants from double-first-class universities accounted for 46.4% of the sample (*n* = 247), and participants from non-double-first-class universities accounted for 53.6% (*n* = 285). In terms of disciplinary categories, 61.1% were from humanities and social sciences (*n* = 325), and 38.9% were from science and engineering (*n* = 207). Participation was voluntary. All data were collected anonymously and treated confidentially.

### 3.2 Measures

Work alienation. Work alienation was measured using an adapted version of a work alienation scale previously used in Chinese organizational research (Long et al., 2014). The original scale contained 26 items and captured five dimensions: normlessness, powerlessness, social alienation, meaninglessness, and self-estrangement. Responses were rated on a seven-point Likert scale ranging from 1 = strongly disagree to 7 = strongly agree, with higher scores indicating higher levels of work alienation.

The work-alienation scale was developed and administered in Chinese. Before the formal survey, terminology was reviewed by an expert panel and the questionnaire underwent cognitive pretesting and pilot testing. During measurement evaluation, before hypothesis testing, two reverse-scored items were removed for weak performance: original Item 15 (social alienation; *λ* = 0.38, CITC = 0.24) and original Item 21 (powerlessness; λ = 0.41, CITC = 0.27). The retained subscales remained reliable and conceptually covered their intended domains; complete item wording and diagnostics are reported in Supplementary Table S2.

Adverse job characteristics. Adverse job characteristics were measured using an adapted 18-item job-characteristics scale previously used in Chinese teacher research (Gan et al., 2006). The scale included four dimensions: lack of support and fairness, perceived busyness, interpersonal strain and conflict, and self-bias/perceived prejudice. Responses were rated on a five-point Likert scale ranging from 1 = completely disagree to 5 = completely agree, with higher scores indicating more negative perceptions of job characteristics.

The source dimension labelled “self-bias/perceived prejudice” is retained for transparency but is operationally interpreted as perceived diminished professional standing, combining self-devaluation with perceived organizational marginalization rather than objectively verified discrimination.

Perceived organizational environment. Perceived organizational environment was measured using a six-item scale adapted for the context of young university teachers (Liu, 2017). The scale assessed teachers’ perceptions of workplace atmosphere, support, affirmation, inspiration, academic opportunities, and reward. Responses were rated on a five-point Likert scale ranging from 1 = completely disagree to 5 = completely agree, with higher scores indicating a more positive perceived organizational environment.

### 3.3 Analytic strategy

Data screening, descriptive statistics, reliability analyses, correlation analyses, hierarchical regression analyses, and moderation analyses were conducted using SPSS and Python-based statistical procedures. Confirmatory factor analyses were conducted using Mplus 8.3 with robust maximum likelihood estimation (MLR). Because the study variables were measured using five- or seven-point Likert-type items and showed no severe univariate non-normality, the items were treated as approximately continuous. Model fit was evaluated using χ^2^, df, χ^2^/df, CFI, TLI, RMSEA, and SRMR. CFA was used to assess the measurement structure, whereas the substantive hypotheses were tested using composite-score regression models.

To assess measurement validity, Cronbach’s alpha, composite reliability (CR), and average variance extracted (AVE) were calculated. Convergent validity was assessed primarily through CR and AVE. Discriminant validity was assessed using the Fornell–Larcker criterion (Fornell and Larcker, 1981) and the heterotrait–monotrait ratio of correlations (HTMT; Henseler et al., 2015). Because adverse job characteristics and work alienation are conceptually close constructs, their discriminant validity was interpreted cautiously and was further evaluated through construct correlations and HTMT values.

The focal scale items had no missing values, and therefore no imputation was required for the main analyses. Normality was assessed using skewness and kurtosis. The focal composite variables showed no severe departure from normality. After item deletion, skewness ranged from −0.502 to 0.493, and kurtosis ranged from −0.800 to −0.320.

Because all focal variables were self-reported at one time point, common-method variance was considered explicitly (Podsakoff et al., 2003). Harman’s first unrotated factor accounted for 47.83% of variance and was treated only as a descriptive screen. A one-factor CFA fit substantially worse than the multidimensional model, but this does not rule out method variance. Procedural remedies included anonymity, confidentiality assurances, separation of predictor and outcome sections, and mixed item wording; no marker-variable or latent-method-factor design was available.

To examine the unique association of each job-characteristic dimension with work alienation, we estimated an integrated model in which all four dimensions of adverse job characteristics were entered simultaneously. Background characteristics were controlled, including gender, age, marital status, institutional level, academic rank, discipline, administrative role, and annual household income. The moderation hypothesis was tested using composite-score hierarchical regression. Adverse job characteristics and perceived organizational environment were mean-centered before creating the interaction term. This approach tests whether the observed composite relationship between adverse job characteristics and work alienation varies by perceived organizational environment. It should therefore be interpreted as a composite-score moderation model rather than a latent interaction SEM. Significant interactions were interpreted using simple slope analysis at one standard deviation above and below the mean of perceived organizational environment.

The 532 teachers were nested within eight universities (41–95 respondents per institution). Unconditional random-intercept models indicated non-negligible clustering: the ICC was 0.180 for work alienation and ranged from 0.124 to 0.175 for the other principal variables. Because only eight clusters were available, primary inference used CR2 bias-reduced cluster-robust standard errors with Satterthwaite-adjusted degrees of freedom, supplemented by wild cluster bootstrap tests (5,000 replications; Webb six-point weights). An exploratory random-intercept multilevel model was used only as a sensitivity analysis (Table 1).

**Table 1:** Institutional clustering of the principal study variables.

Variable	Between-university variance	Within-university variance	ICC	95% CI
Work alienation	0.158	0.719	0.180	[0.120, 0.250]
Adverse job characteristics, composite	0.142	0.736	0.162	[0.104, 0.235]
Lack of support and fairness	0.168	0.792	0.175	[0.114, 0.248]
Perceived busyness	0.098	0.694	0.124	[0.075, 0.189]
Interpersonal strain and conflict	0.189	0.911	0.172	[0.112, 0.244]
Self-bias/perceived prejudice	0.151	0.871	0.148	[0.092, 0.218]
Perceived organizational environment	0.135	0.786	0.146	[0.090, 0.216]

ICC, intraclass correlation coefficient. Confidence intervals were estimated from separate unconditional random-intercept models using 5,000 parametric bootstrap replications. With only eight institutions, the variance components and confidence intervals should be interpreted cautiously.

## 4 Results

### 4.1 Measurement validation

The revised 24-item work-alienation scale was evaluated using a correlated five-factor first-order CFA. The model showed acceptable fit: χ^2^ = 945.68, df = 242, CFI = 0.931, TLI = 0.921, RMSEA = 0.074, 90% CI [0.069, 0.079], and SRMR = 0.056 (Tables 2, 3).

**Table 2:** Reliability and convergent validity.

Construct/dimension	Indicators	Cronbach’s α	CR	AVE
Work alienation (higher-order)	5 dimensions	0.917	0.937	0.748
Normlessness	6 items	0.931	0.946	0.746
Powerlessness	6 items	0.922	0.939	0.728
Social alienation	5 items	0.842	0.887	0.631
Meaninglessness	3 items	0.790	0.794	0.563
Self-estrangement	4 items	0.937	0.955	0.841
Adverse job characteristics (higher-order)	4 dimensions	0.843	0.929	0.767
Lack of support and fairness	6 items	0.927	0.943	0.734
Perceived busyness	5 items	0.882	0.915	0.683
Interpersonal strain and conflict	3 items	0.885	0.929	0.813
Self-bias/perceived prejudice	4 items	0.840	0.894	0.680
Perceived organizational environment	6 items	0.932	0.947	0.748

CR, composite reliability; AVE, average variance extracted. For the higher-order work-alienation and adverse-job-characteristics constructs, CR and AVE were calculated from the standardized second-order loadings of the corresponding first-order dimensions.

**Table 3:** Fornell-Larcker assessment.

Construct	AVE	1	2	3
1. Work alienation	0.748	0.865		
2. Adverse job characteristics	0.767	0.847	0.876	
3. Perceived organizational environment	0.748	−0.592	−0.474	0.865

*N* = 532. AVE, average variance extracted. Diagonal values are square roots of AVE, and off-diagonal values are construct-level correlations. For the higher-order WA and AJC constructs, AVE was calculated from the standardized second-order loadings of the corresponding first-order dimensions.

Given the high WA-AJC correlation (r = 0.847), discriminant validity was evaluated using the combined measurement model, alternative models, HTMT, the Fornell-Larcker criterion, item-content review, and sensitivity analyses. The hypothesized multidimensional model fit acceptably (CFI = 0.918, TLI = 0.912, RMSEA = 0.053, SRMR = 0.049) and better than the tested merged alternatives (Table 4). HTMT for WA-AJC was 0.886, 95% CI [0.861, 0.910], indicating borderline fixed-threshold evidence; the corresponding values for WA-POE and AJC-POE were 0.631 and 0.507. AVE/√AVE values were 0.748/0.865 for WA, 0.767/0.876 for AJC, and 0.748/0.865 for POE, satisfying the Fornell-Larcker criterion. Nevertheless, content review identified overlap in colleague support/isolation, self-evaluation, organizational belonging, interpersonal difficulties, and effort-reward imbalance. Excluding the self-bias/perceived-prejudice dimension or six overlapping items reduced the WA-AJC correlation to 0.782 and 0.761 and HTMT to 0.820 and 0.798, respectively (Supplementary Table S3). Thus, the evidence supports treating the constructs separately but is not unequivocal; their association may partly reflect conceptual overlap and common-method influences.

**Table 4:** Integrated model predicting work alienation.

Predictor	β	SE	t	*p*
Lack of support and fairness	0.619	0.035	17.777	< 0.001
Perceived busyness	0.122	0.030	4.083	< 0.001
Interpersonal strain and conflict	0.084	0.031	2.721	0.007
Self-bias / perceived prejudice	0.158	0.028	5.578	< 0.001

R^2^ = 0.767, adjusted R^2^ = 0.761, F = 142.103, p < 0.001. Background characteristics were controlled.

The revised 24-item work-alienation scale was evaluated using a correlated five-factor first-order CFA. The model showed acceptable fit: χ^2^ = 945.68, df = 242, CFI = 0.931, TLI = 0.921, RMSEA = 0.074, 90% CI [0.069, 0.079], and SRMR = 0.056 (Table 5).

**Table 5:** Comparison of the hypothesized and alternative measurement models.

Model	Model specification	CFI	TLI	RMSEA	SRMR	AIC	BIC	ΔCFI	ΔRMSEA
M1	Five WA dimensions - > second-order WA; four AJC dimensions - > second-order AJC; POE separate.	0.918	0.912	0.053	0.049	48,245.2	48,892.7	-	-
M2	Nine WA/AJC dimensions - > 1 second-order negative-work-experience factor; POE separate.	0.841	0.833	0.074	0.071	49,621.8	50,253.4	−0.077	+0.021
M3a	Six LSF items + six reverse-oriented POE items - > one organizational-evaluation factor; three remaining AJC dimensions - > second-order AJC.	0.864	0.857	0.079	0.073	49,894.5	50,528.1	−0.054	+0.026
M3b	Four AJC dimensions and POE specified under a broader organizational-evaluation construct; WA separate.	0.815	0.807	0.081	0.078	50,203.1	50,820.9	−0.103	+0.028
M4	All 48 items loaded on one general factor.	0.668	0.653	0.114	0.121	53,912.4	54,508.7	−0.250	+0.061

*N* = 532. WA, work alienation; AJC, adverse job characteristics; POE = perceived organizational environment. All models were estimated using MLR in Mplus 8.3. Some alternatives changed both the composition of the higher-order AJC factor and the status of POE and were therefore treated as theoretically motivated non-nested alternatives. Comparisons were based on the overall pattern of fit indices and information criteria, not unadjusted chi-square differences. ΔCFI, CFI_alternative - CFI_M1; ΔRMSEA, RMSEA_alternative - RMSEA_M1.

### 4.2 Integrated model of adverse job characteristics and work alienation

An integrated model was estimated in which all four dimensions of adverse job characteristics were entered simultaneously to examine their unique associations with work alienation, with background characteristics controlled. The ordinary-regression estimates are reported in Table 6; primary coefficient inference is based on the cluster-adjusted analyses reported below.

**Table 6:** HTMT ratios with bootstrap 95% confidence intervals.

Construct pair	HTMT	Bootstrap 95% CI	Interpretation
Work alienation - adverse job characteristics	0.886	[0.861, 0.910]	Borderline fixed-threshold evidence; CI does not include 1.00
Work alienation - perceived organizational environment	0.631	[0.588, 0.674]	Discriminant validity supported
Adverse job characteristics - perceived organizational environment	0.507	[0.455, 0.559]	Discriminant validity supported

HTMT, heterotrait-monotrait ratio of correlations. Confidence intervals were estimated using 5,000 individual-level bootstrap replications and did not reproduce teacher clustering within universities. The WA-AJC estimate was interpreted together with the Fornell-Larcker assessment and item-content review.

The integrated ordinary model explained 76.7% of the variance in work alienation, adjusted R^2^ = 0.761, *F* = 142.103, *p* < 0.001. The VIF values ranged from 1.06 to 2.70, indicating no serious multicollinearity. These statistics describe ordinary-regression model fit; primary coefficient inference is based on the cluster-adjusted results in Table 7.

**Table 7:** Cluster-adjusted regression results for the main and moderating associations.

Model/predictor	B	Standardized *β*	CR2 SE	Satterthwaite df	CR2 p	CR2 95% CI	Wild-bootstrap p
Integrated main-effects model							
Lack of support and fairness	0.601	0.619	0.041	6.8	<0.001	[0.503, 0.699]	0.002
Perceived busyness	0.115	0.122	0.035	6.5	0.015	[0.031, 0.199]	0.006
Interpersonal strain and conflict	0.079	0.084	0.036	6.3	0.069	[−0.008, 0.166]	0.052
Self-bias/perceived prejudice	0.150	0.158	0.033	6.7	0.003	[0.071, 0.229]	0.004
Moderation model							
Adverse job characteristics	0.741	0.745	0.030	6.9	<0.001	[0.670, 0.812]	0.002
Perceived organizational environment	−0.208	−0.221	0.033	6.5	<0.001	[−0.287, −0.129]	0.004
AJC × POE	−0.032	−0.034	0.021	6.2	0.177	[−0.083, 0.019]	0.148

*N* = 532 teachers nested within eight universities. B denotes the unstandardized regression coefficient. AJC, adverse job characteristics; POE, perceived organizational environment. CR2 denotes the bias-reduced cluster-robust variance estimator with Satterthwaite-adjusted degrees of freedom. Wild cluster bootstrap tests used 5,000 replications and Webb six-point weights. Both models adjusted for gender, age, marital status, education, academic rank, discipline, administrative role, institutional category, and income. Complete control-variable estimates are reported in Supplementary Table S4.

A two-step hierarchical regression entered nine background variables in Step 1 and the four adverse-job-characteristic dimensions in Step 2. Step 1 explained 6.1% of the variance in work alienation; the final ordinary model explained 76.7% (ΔR^2^ = 0.706), with VIFs of 1.06–2.70 (Table 8). These statistics describe model fit and incremental variance; coefficient inference is based on the cluster-adjusted results in Table 7. Complete control-variable estimates are provided in Supplementary Table S4.

**Table 8:** Corrected hierarchical model summary.

Model	R^2^	Adjusted R^2^	F (df)	ΔR^2^	ΔF (df)	Incremental f^2^
Step 1: nine controls	0.061	0.045	3.77 (9, 522), p < 0.001	—	—	—
Step 2: controls + four AJC dimensions	0.767	0.761	131.17 (13, 518), p < 0.001	0.706	392.39 (4, 518), p < 0.001	3.03

*N* = 532. AJC = adverse job characteristics. These are ordinary-regression model-summary statistics. The focal coefficient inferences are evaluated using the cluster-adjusted results in Table 7.

Cluster-adjusted inference supported positive associations of lack of support and fairness, perceived busyness, and self-bias/perceived prejudice with work alienation, whereas interpersonal strain and conflict was not robust (CR2 *p* = 0.069; wild-bootstrap *p* = 0.052). Perceived organizational environment remained negatively associated with work alienation. The AJC × POE interaction was not significant after cluster adjustment (B = −0.032, standardized *β* = −0.034, CR2 *p* = 0.177; wild-bootstrap *p* = 0.148); therefore, Hypothesis 3 was not supported. The exploratory multilevel analysis yielded the same substantive conclusion (Table 7).

### 4.3 Background characteristics and work alienation

Teachers’ background characteristics were then examined in relation to overall work alienation and its five dimensions. The results suggest that some background characteristics were associated with specific dimensions of work alienation, although these associations were not uniform across all indicators. Marital status was negatively associated with social alienation and with the overall work alienation score, indicating that married teachers reported somewhat lower levels of alienation on these measures. Institutional level was positively associated with normlessness, powerlessness, and the overall alienation score, suggesting that teachers in non-double-first-class institutions may face greater vulnerability on some dimensions. By contrast, gender, academic discipline, and most rank categories showed limited or inconsistent associations. These findings indicate that background characteristics matter, but their explanatory role appears more selective and less consistent than that of adverse job characteristics and organizational environment.

### 4.4 Moderating role of perceived organizational environment

Adverse job characteristics and perceived organizational environment were mean-centered before their interaction was computed. In the ordinary regression, the interaction was very small (B = −0.032, standardized *β* = −0.034, *p* = 0.047; ΔR^2^ = 0.001, f^2^ = 0.005). More importantly, it was not significant after accounting for institutional clustering (CR2 *p* = 0.177; wild-bootstrap *p* = 0.148). Hypothesis 3 was therefore not supported under the primary inference. Perceived organizational environment is interpreted as a negative correlate of work alienation rather than as a reliably demonstrated buffer (Table 9)

**Table 9:** Moderation model predicting work alienation.

Predictor	β	SE	t	p
Adverse job characteristics	0.745	0.024	30.659	< 0.001
Perceived organizational environment	−0.221	0.026	−8.386	< 0.001
Adverse job characteristics × Perceived organizational environment	−0.034	0.017	−1.978	0.048

Ordinary-regression estimates are shown for descriptive comparability. Primary inference for the moderation term is based on the cluster-adjusted results in Table 7.

Cluster-adjusted inference supported positive associations of lack of support and fairness, perceived busyness, and self-bias/perceived prejudice with work alienation, whereas interpersonal strain and conflict was not robust (CR2 *p* = 0.069; wild-bootstrap *p* = 0.052). Perceived organizational environment remained negatively associated with work alienation. The AJC × POE interaction was not significant after cluster adjustment (B = −0.032, standardized β = −0.034, CR2 *p* = 0.177; wild-bootstrap *p* = 0.148); therefore, Hypothesis 3 was not supported. The exploratory multilevel analysis yielded the same substantive conclusion (Table 7).

## 5 Discussion

Accounting for institutional clustering qualified the findings: three adverse-job-characteristic dimensions remained positively associated with work alienation, whereas interpersonal strain and conflict was not robust. Perceived organizational environment showed a negative individual-level association with work alienation, but no robust buffering interaction was detected. The findings represent statistical associations rather than causal effects. Lack of support and fairness, perceived busyness, and self-bias/perceived prejudice were positively associated with work alienation after cluster adjustment, while perceived organizational environment was negatively associated with work alienation. Reverse and reciprocal interpretations remain plausible because alienated teachers may also evaluate their work conditions more negatively.

### 5.1 Theoretical implications

This study makes several theoretical contributions to the literature on work alienation and academic work. First, it enriches work alienation research by extending the construct to the context of young university teachers in Chinese higher education. Prior research has often examined alienation in general organizational settings or has focused on related but distinct outcomes such as burnout, job dissatisfaction, turnover intention, and work stress. Although these constructs are important, they do not fully capture the deeper rupture between teachers and the meaning, autonomy, belonging, and self-identification associated with academic work. By focusing on work alienation, this study shows that young academics may remain formally productive while experiencing a weakened psychological connection to teaching, research, collegial life, and the broader purposes of the academic profession. This distinction is especially important in performance-oriented higher education systems, where compliance with evaluation requirements may coexist with declining professional identification. This finding confirms prior work that treats alienation and anomie as consequences of deteriorating academic work conditions, but it also extends that work by specifying alienation as a multidimensional psychological state rather than as general dissatisfaction or exit intention. In this sense, the study differs from Watermeyer et al. (2024), who emphasize academic anomie and sectoral departure, by showing how alienation can be present among teachers who remain within the institution and continue to perform academic roles.

The strong WA-AJC association should be interpreted cautiously. Although alternative measurement models and sensitivity analyses support retaining separate constructs, some item content overlaps and all focal measures were self-reported at the same time point. The association may therefore reflect both substantive proximity and shared-method or reciprocal processes and should not be interpreted as an independent causal effect. Future studies should use temporally separated, multi-source, and alternative measurement designs.

Second, the cluster-adjusted results differentiate among the adverse-job-characteristic dimensions. Lack of support and fairness, perceived busyness, and self-bias/perceived prejudice remained positively associated with work alienation, whereas interpersonal strain and conflict was not robust after institutional clustering was taken into account. This pattern suggests that the organization, evaluation, and recognition of academic work are especially salient correlates of alienation, while dimension-specific conclusions should be drawn cautiously.

Third, perceived organizational environment showed a negative individual-level association with work alienation, but the proposed buffering interaction was not supported under the primary cluster-adjusted inference. The moderation hypothesis was not supported under the primary cluster-adjusted inference. The nominal ordinary-regression interaction was very small and unstable across inferential methods; therefore, it is not interpreted as evidence of a reliable buffering mechanism.

Fourth, this study offers a more context-sensitive understanding of academic work in contemporary China. Young university teachers in China often work within competitive appointment arrangements, intensified performance evaluation, and uncertain career-development pathways. Recent studies of Chinese early-career academics have described how tenure-track or “up-or-out” arrangements, resource competition, and normalized overwork can produce insecurity, anxiety, and involution-like adaptation (Si, 2023; Yin and Mu, 2023). The present study complements these qualitative accounts with quantitative evidence showing that alienation is associated not only with heavy workload but also with perceived unfairness, inadequate support, interpersonal strain, and perceived bias. In this sense, the study helps explain how structural features of Chinese higher education governance are translated into teachers’ everyday psychological experience.

Finally, the findings also contribute to international debates on academic identity and changing higher education work. The mechanisms identified here are not limited to China, even though they are shaped by the Chinese institutional context. Across higher education systems, academics increasingly face intensified performance demands, managerial evaluation, unstable career expectations, and tensions between professional autonomy and institutional accountability. Studies of academic anomie, faculty alienation, academic identity, and job demands/resources similarly suggest that deteriorating work environments can weaken meaning, belonging, and professional commitment (Bougherza et al., 2024; Marques et al., 2024; Naidoo-Chetty and du Plessis, 2021; Watermeyer et al., 2024). Thus, the study positions Chinese young academics as a contextually specific but theoretically informative case for understanding the changing nature of academic labor. These findings also connect to academic identity research. Marques et al. (2024) show that higher education change reshapes academic identities; the present study adds that such identity pressures may become alienating when teachers experience weak recognition, strained relations, or limited professional autonomy. Thus, the study positions work alienation as a bridge between research on academic identity and research on academic work conditions.

Common-method variance cannot be excluded and may have inflated associations among the self-reported constructs. The one-factor comparison only shows that a single general factor is inadequate; it does not establish absence of method bias. Future studies should use temporally separated, multi-source, or marker-variable designs.

### 5.2 Practical implications

The findings also have several practical implications. First, universities should treat work alienation as an organizational and governance issue rather than as a purely individual problem. If young teachers experience alienation mainly because work is perceived as unfair, unsupported, overly busy, relationally strained, or biased, then interventions that focus only on individual resilience or emotional adjustment are insufficient. Universities need to examine how evaluation rules, promotion criteria, workload allocation, resource distribution, and administrative procedures shape teachers’ sense of meaning, control, and professional recognition. In particular, transparent evaluation standards, timely feedback, fair access to research and teaching resources, and credible appeal mechanisms can help reduce perceptions of unfairness and lack of support.

Second, universities should reduce the alienating effects of chronic busyness by improving workload governance. Young teachers often face simultaneous demands in teaching, research, student supervision, administrative service, and performance assessment. When these demands accumulate without coordination, academic work can become fragmented and difficult to connect to professional purpose. Universities should therefore monitor workload distribution, reduce unnecessary administrative tasks, coordinate teaching and research expectations, and provide protected time for academic development. Such measures are especially important for early-career teachers who are still building research capacity, teaching confidence, and institutional networks.

Third, institutions should strengthen the relational and developmental environment surrounding young academics. Although interpersonal strain and conflict was not robust after cluster adjustment, self-bias/perceived prejudice remained positively associated with work alienation, underscoring the importance of recognition, belonging, mentoring, inclusive communication, and fair access to professional opportunities.

Fourth, although the proposed moderation was not supported after institutional clustering was taken into account, a more positive perceived organizational environment remained associated with lower work alienation. Universities can therefore strengthen concrete forms of organizational support, including accessible mentorship, fair performance appraisal, recognition of teaching and service, support for grant and publication development, and clear career-development pathways.

Finally, policy makers and university leaders should recognize that work alienation may remain hidden in performance-oriented systems. Young teachers may continue to publish, teach, and meet assessment targets while feeling detached from the meaning and autonomy of academic work. Therefore, universities should not rely only on output indicators to evaluate the health of academic work. They should also attend to teachers’ perceptions of fairness, support, recognition, belonging, and professional sustainability. Reducing work alienation requires institutional efforts to make academic work not only productive but also meaningful, autonomous, relationally supportive, and professionally sustainable.

## 6 Conclusion

Several adverse job characteristics remained positively associated with work alienation after cluster adjustment, and perceived organizational environment was negatively associated with work alienation. However, the AJC × POE interaction was not significant; the findings therefore support a direct association with perceived organizational environment, not the hypothesized moderation. Using cross-sectional survey data, this study identified associations between perceived work conditions, perceived organizational environment, and work alienation; causal and temporal interpretations require longitudinal or experimental evidence.

## 7 Limitations and further research

First, the cross-sectional design does not establish temporal order or causality among adverse job characteristics, perceived organizational environment, and work alienation. Reverse causation, reciprocal processes, common-method variance, negative affectivity, and omitted variables remain plausible. Future research should prioritize longitudinal or repeated-measures designs and, where feasible, multi-source, quasi-experimental, or intervention evidence.

Second, purposive selection of eight universities and convenience sampling of faculty may have introduced institutional gatekeeper, nonresponse, and self-selection bias. Regional coverage was uneven and no quotas, sampling weights, or post-stratification were used. Generalization to all young university teachers in China should therefore be cautious; future studies should use larger, more balanced probability-based or stratified samples.

Third, only eight universities were included. Although CR2 and wild cluster bootstrap procedures were used, inference with so few clusters remains uncertain, and institution-level variance cannot be estimated precisely. The AJC × POE term represents an individual-level interaction between teachers’ perceptions, not a cross-level moderation effect. Future studies should include substantially more institutions and determine the required cluster count through design-specific power analysis or simulation.

Because the final 24-item scale was modified and evaluated in the same analytic sample, its psychometric stability has not been independently established. Replication should evaluate its factor structure, reliability, invariance, and criterion validity in independent samples.

Common-method variance cannot be excluded and may have inflated associations among the self-reported constructs. The one-factor comparison only shows that a single general factor is inadequate; it does not establish absence of method bias. Future studies should use temporally separated, multi-source, or marker-variable designs.

## Data Availability

The raw data supporting the conclusions of this article will be made available by the authors, without undue reservation.

## References

[ref1] Bakker A. B. Demerouti E. (2007). The job demands-resources model: state of the art. J. Manager. Psychol. 22, 309–328. doi: 10.1108/02683940710733115

[ref2] Bakker A. B. Hakanen J. J. Demerouti E. Xanthopoulou D. (2007). Job resources boost work engagement, particularly when job demands are high. J. Educ. Psychol. 99, 274–284. doi: 10.1037/0022-0663.99.2.274

[ref3] Banai M. Reisel W. D. (2007). The influence of supportive leadership and job characteristics on work alienation: a six-country investigation. J. World Bus. 42, 463–476. doi: 10.1016/j.jwb.2007.06.007

[ref4] Bougherza R. Azieb S. Abdo Noufal Z. M. Mallek M. Abderrahmane Y. Brachene I. E. . (2024). The internal work environment and job alienation: the case of faculty members. Emerg. Sci. J. 8, 103–117. doi: 10.28991/ESJ-2024-SIED1-07

[ref5] Cohen-Charash Y. Spector P. E. (2001). The role of justice in organizations: a meta-analysis. Organ. Behav. Hum. Decis. Process. 86, 278–321. doi: 10.1006/obhd.2001.2958

[ref6] Cole M. S. Bernerth J. B. Walter F. Holt D. T. (2010). Organizational justice and individuals’ withdrawal: unlocking the influence of emotional exhaustion. J. Manag. Stud. 47, 367–390. doi: 10.1111/j.1467-6486.2009.00864.x

[ref7] Colquitt J. A. Conlon D. E. Wesson M. J. Porter C. O. L. H. Ng K. Y. (2001). Justice at the millennium: a meta-analytic review of 25 years of organizational justice research. J. Appl. Psychol. 86, 425–445. doi: 10.1037/0021-9010.86.3.425, 11419803

[ref8] Dean D. G. (1961). Alienation: its meaning and measurement. Am. Sociol. Rev. 26, 753–758. doi: 10.2307/2090204

[ref9] Demerouti E. Bakker A. B. Nachreiner F. Schaufeli W. B. (2001). The job demands-resources model of burnout. J. Appl. Psychol. 86, 499–512. doi: 10.1037/0021-9010.86.3.499, 11419809

[ref10] Eisenberger R. Fasolo P. Davis-LaMastro V. (1990). Perceived organizational support and employee diligence, commitment, and innovation. J. Appl. Psychol. 75, 51–59. doi: 10.1037/0021-9010.75.1.51

[ref11] Eisenberger R. Huntington R. Hutchison S. Sowa D. (1986). Perceived organizational support. J. Appl. Psychol. 71, 500–507. doi: 10.1037/0021-9010.71.3.500

[ref12] Fornell C. Larcker D. F. (1981). Evaluating structural equation models with unobservable variables and measurement error. J. Mark. Res. 18, 39–50. doi: 10.1177/002224378101800104

[ref13] Gan Y. Wang X. Zhang Y. Zhang Y. (2006). Job Characteristics that Affecting Job Burnout among Secondary School Teachers in China’s Rural Area. Acta Psychologica Sinica, 38, 92–98.

[ref14] Hackman J. R. Oldham G. R. (1976). Motivation through the design of work: test of a theory. Organ. Behav. Hum. Perform. 16, 250–279. doi: 10.1016/0030-5073(76)90016-7

[ref15] Halal Orfali C. Arancibia Muñoz M. L. Riquelme Plaza I. Unda Valenzuela R. (2024). How higher education teachers see their professional identity. Front. Educ. 9:1429847. doi: 10.3389/feduc.2024.1429847

[ref16] Henseler J. Ringle C. M. Sarstedt M. (2015). A new criterion for assessing discriminant validity in variance-based structural equation modeling. J. Acad. Mark. Sci. 43, 115–135. doi: 10.1007/s11747-014-0403-8

[ref17] Humphrey S. E. Nahrgang J. D. Morgeson F. P. (2007). Integrating motivational, social, and contextual work design features: a meta-analytic summary and theoretical extension of the work design literature. J. Appl. Psychol. 92, 1332–1356. doi: 10.1037/0021-9010.92.5.1332 17845089

[ref18] Ji H. Xia K. Wang Y. Li J. Liu J. He L. . (2025). Relationship teachers’ perception of organizational justice, job burnout and organizational citizenship behavior. BMC Psychol. 13:160. doi: 10.1186/s40359-025-02422-8, 39994751 PMC11849375

[ref19] Korumaz M. Kilic G. N. Kocabas I. (2020). School culture as a predictor of teachers’ alienation at schools. Int. Online J. Educ. Sci. 12, 46–62. doi: 10.15345/iojes.2020.03.004

[ref20] Liu L. (2017) Study on the influence of coach’s sense of work alienation on job performance 2nd International Symposium on Business Corporation and Development in South-East and South Asia under B&R Initiative (ISBCD 2017) 289–292. Paris, France: Atlantis Press

[ref21] Long L. Mao P. Zhang Y. Huang S. B. (2014). The effects of paternalistic leadership mediated by perceptions of organisational support on employees’ job detachment (in Chinese). J. Manag. 11, 1150–1157. doi: 10.3969/j.issn.1672-884x.2014.08.008

[ref22] Marques R. M. G. Lopes A. Magalhães A. M. (2024). Academic identities and higher education change: reviewing the evidence. Educ. Res. 66, 228–244. doi: 10.1080/00131881.2024.2334760

[ref23] Ministry of Education (2025) 2024年全国教育事业发展统计剬报 [Statistical Bulletin on national Education Development in 2024] Available online at: http://www.moe.gov.cn/jyb_sjzl/sjzl_fztjgb/202506/t20250611_1193760.html (Accessed August 16, 2026).

[ref24] Ministry of Education National Development and Reform Commission Ministry of Science and Technology Ministry of Industry and Information Technology Ministry of Finance Ministry of Human Resources and Social Security (2025) 教育部等六部门关于加强新时代高校青年教师队伍建设的指导意见 [Guiding Opinions on Strengthening the Development of young University Teachers in the new era] Available online at: http://www.moe.gov.cn/srcsite/A10/s7151/202511/t20251105_1419084.html (Accessed August 16, 2026).

[ref25] Naidoo-Chetty M. du Plessis M. (2021). Job demands and job resources of academics in higher education. Front. Psychol. 12:631171. doi: 10.3389/fpsyg.2021.631171, 34248738 PMC8260971

[ref26] O’Meara K. Lennartz C. J. Kuvaeva A. Jaeger A. Misra J. (2019). Department conditions and practices associated with faculty workload satisfaction and perceptions of equity. J. High. Educ. 90, 744–772. doi: 10.1080/00221546.2019.1584025

[ref27] Piano M. Jarden R. J. Hastings-Ison T. Lawford B. J. Diemer K. Hui F. . (2024). Exploring early- and mid-career academic work wellbeing challenges through a diversity and inclusion lens. BMC Med. Educ. 24:1048. doi: 10.1186/s12909-024-05967-1, 39334041 PMC11428564

[ref28] Podsakoff P. M. MacKenzie S. B. Lee J.-Y. Podsakoff N. P. (2003). Common method biases in behavioral research: a critical review of the literature and recommended remedies. J. Appl. Psychol. 88, 879–903. doi: 10.1037/0021-9010.88.5.879, 14516251

[ref29] Rhoades L. Eisenberger R. (2002). Perceived organizational support: a review of the literature. J. Appl. Psychol. 87, 698–714. doi: 10.1037/0021-9010.87.4.698, 12184574

[ref30] Salancik G. R. Pfeffer J. (1978). A social information processing approach to job attitudes and task design. Adm. Sci. Q. 23, 224–253. doi: 10.2307/2392563, 10307892

[ref31] Seeman M. (1959). On the meaning of alienation. Am. Sociol. Rev. 24, 783–791. doi: 10.2307/2088565

[ref32] Si J. (2023). No other choices but involution: understanding Chinese young academics in the tenure track system. J. High. Educ. Policy Manag. 45, 53–67. doi: 10.1080/1360080X.2022.2115332

[ref33] Stratford E. Watson P. Paull B. (2024). What impedes and enables flourishing among early career academics? High. Educ. 88, 259–277. doi: 10.1007/s10734-023-01115-8

[ref34] Sulu S. Ceylan A. Kaynak R. (2010). Work alienation as a mediator of the relationship between organizational injustice and organizational commitment: implications for healthcare professionals. Int. J. Bus. Manag. 5, 27–38. doi: 10.5539/ijbm.v5n8p27

[ref35] Wang T. Wang W. Dai K. (2025). What organizational supports matter in scholar development? An investigation of early career academics’ experiences in China. Asia Pac. Educ. Rev., 1–14. doi: 10.1007/s12564-025-10054-1

[ref36] Watermeyer R. Bolden R. Knight C. Crick T. (2024). Academic anomie: implications of the “great resignation” for leadership in post-COVID higher education. High. Educ. 89, 1215–1233. doi: 10.1007/s10734-024-01268-0

[ref37] Yin Y. M. Mu G. M. (2023). Thriving in the neoliberal academia without becoming its agent? Sociologising resilience with an early career academic and a mid-career researcher. High. Educ. 86, 65–80. doi: 10.1007/s10734-022-00901-0

