# Supplementary material for: Work alienation among young university teachers in China
Source: Front Psychol. 2026 Aug 28;17:1845561. doi: 10.3389/fpsyg.2026.1845561 (PMC13562015; doi:10.3389/fpsyg.2026.1845561)
Supplement: Supplementary file 1 [file Data_Sheet_1.PDF]

Supplementary Materials

Work Alienation among Young University Teachers in China

Supplementary Table S1. Complete Standardized Factor Loadings, Standard Errors, 95% Confidence Intervals, and Residual Variances for All Measurement Items

Note. This table reports complete item-level and higher-order results from the final combined measurement model estimated using robust maximum likelihood (MLR) in Mplus 8.3. All loadings are statistically significant at $p < .001$. Standardized residual variances for first-order indicators were calculated as $1 - \lambda^2$ and are derived values rather than direct Mplus estimates. For second-order dimensions, R^2 is reported as the explained variance. N = 532.

Part A: Work Alienation – First-Order Loadings (Items → Dimensions)

Item Code	Dimension	Abbreviated Item Content	λ	Robust SE	95% CI	Standardized Residual Variance
WA1	Normlessness	The work assigned makes me confused	0.86	0.023	[0.82, 0.91]	0.260
WA3	Normlessness	Only by maintaining good interpersonal relationships can one get promoted	0.87	0.022	[0.83, 0.91]	0.243
WA4	Normlessness	Rewards and punishments are unclear	0.88	0.021	[0.84, 0.92]	0.226
WA5	Normlessness	To get promoted, one must maintain good relationships with leaders	0.86	0.023	[0.82, 0.91]	0.260
WA6	Normlessness	My work is heavy, but I do not receive corresponding rewards	0.85	0.024	[0.80, 0.90]	0.278
WA11	Normlessness	Promotion basically depends on relationships	0.85	0.024	[0.80, 0.90]	0.278
WA7	Powerlessness	Work is routine and lacks creativity	0.85	0.024	[0.80, 0.90]	0.278
WA8	Powerlessness	The greatest meaning of my work is just this salary	0.86	0.023	[0.82, 0.91]	0.260
WA9	Powerlessness	To maintain my livelihood, I have to continue	0.87	0.022	[0.83, 0.91]	0.243
WA10	Powerlessness	I do not like my work; this job cannot realize my value	0.87	0.022	[0.83, 0.91]	0.243

Item Code	Dimension	Abbreviated Item Content	λ	Robust SE	95% CI	Standardized Residual Variance
WA12	Powerlessness	My work can showcase my talents well (R)	0.84	0.025	[0.79, 0.89]	0.294
WA2	Powerlessness	I often have to do things I do not want to do	0.85	0.024	[0.80, 0.90]	0.278
WA13	Social alienation	I get along very well with my colleagues (R)	0.80	0.028	[0.75, 0.85]	0.360
WA15	Social alienation	Disagreements do not affect our relationships (R)	0.79	0.029	[0.73, 0.85]	0.376
WA17	Social alienation	I often cannot get sincere help from colleagues	0.82	0.027	[0.77, 0.87]	0.328
WA18	Social alienation	Sometimes I feel like I am alone in the institution	0.78	0.030	[0.72, 0.84]	0.392
WA25	Social alienation	I often feel lonely at work	0.79	0.029	[0.73, 0.85]	0.376
WA19	Meaninglessness	Regarding promotion, I think the prospects are dim	0.78	0.030	[0.72, 0.84]	0.392
WA20	Meaninglessness	Current busy work lays foundation for future (R)	0.75	0.033	[0.69, 0.81]	0.438
WA22	Meaninglessness	Overcoming difficulties is one of the great pleasures (R)	0.72	0.035	[0.65, 0.79]	0.482
WA23	Self-estrangement	I always feel like I am a failure	0.93	0.017	[0.90, 0.96]	0.135
WA24	Self-estrangement	I often doubt my work ability	0.92	0.018	[0.88, 0.96]	0.154
WA26	Self-estrangement	No matter how hard I try, I cannot get others' recognition	0.91	0.019	[0.87, 0.95]	0.172
WA16	Self-estrangement	I am just an ordinary member; as long as I do not make mistakes	0.91	0.019	[0.87, 0.95]	0.172

Part B: Work Alienation – Second-Order Loadings (Dimensions → Work Alienation Factor)

First-Order Dimension	λ	Robust SE	95% CI	R^2 (Explained Variance)
Normlessness → Work Alienation	0.91	0.019	[0.87, 0.95]	0.828
Powerlessness → Work Alienation	0.90	0.020	[0.86, 0.94]	0.810
Social alienation → Work Alienation	0.84	0.025	[0.79, 0.89]	0.706
Meaninglessness → Work Alienation	0.80	0.028	[0.75, 0.85]	0.640
Self-estrangement → Work Alienation	0.87	0.022	[0.83, 0.91]	0.757

Note. R^2 indicates the proportion of variance in each first-order dimension explained by the second-order work-alienation factor.

Part C: Adverse Job Characteristics – First-Order Loadings (Items → Dimensions)

Item Code	Dimension	Abbreviated Item Content	λ	Robust SE	95% CI	Standardized Residual Variance
AJC1	Lack of support/fairness	I do not feel support from leaders	0.88	0.021	[0.84, 0.92]	0.226
AJC2	Lack of support/fairness	I do not feel support from colleagues	0.87	0.022	[0.83, 0.91]	0.243
AJC3	Lack of support/fairness	I encounter "exploitation" from colleagues or leaders	0.86	0.023	[0.82, 0.91]	0.260
AJC4	Lack of support/fairness	I do not feel support in academic publication and project application	0.85	0.024	[0.80, 0.90]	0.278
AJC5	Lack of support/fairness	I often cannot find research collaborators	0.85	0.024	[0.80, 0.90]	0.278
AJC6	Lack of support/fairness	Some leaders create obstacles for my work	0.84	0.025	[0.79, 0.89]	0.294
AJC7	Perceived busyness	This job leaves me almost no personal time	0.85	0.024	[0.80, 0.90]	0.278
AJC8	Perceived busyness	I feel busy almost every day	0.84	0.025	[0.79, 0.89]	0.294
AJC9	Perceived busyness	I hardly know when I am truly "off work"	0.82	0.027	[0.77, 0.87]	0.328

Item Code	Dimension	Abbreviated Item Content	λ	Robust SE	95% CI	Standardized Residual Variance
AJC10	Perceived busyness	My efforts far exceed what I get	0.82	0.027	[0.77, 0.87]	0.328
AJC11	Perceived busyness	What I have to do each day is always more than I imagined	0.80	0.028	[0.75, 0.85]	0.360
AJC12	Interpersonal strain/conflict	Dealing with interpersonal relationships makes me tired and annoyed	0.92	0.018	[0.88, 0.96]	0.154
AJC13	Interpersonal strain/conflict	Interpersonal matters often affect my work mood	0.90	0.020	[0.86, 0.94]	0.190
AJC14	Interpersonal strain/conflict	I hope there is less internal friction from interpersonal relationships	0.88	0.021	[0.84, 0.92]	0.226
AJC15	self-bias/perceived prejudice	I think I am not diligent enough	0.85	0.024	[0.80, 0.90]	0.278
AJC16	self-bias/perceived prejudice	I think my ability is insufficient	0.83	0.026	[0.78, 0.88]	0.311
AJC17	self-bias/perceived prejudice	I think the institution could replace me at any time	0.82	0.027	[0.77, 0.87]	0.328
AJC18	self-bias/perceived prejudice	I think I am not a real member of the institution	0.80	0.028	[0.75, 0.85]	0.360

Part D: Adverse Job Characteristics – Second-Order Loadings (Dimensions → AJC Factor)

First-Order Dimension	λ	Robust SE	95% CI	R^2 (Explained Variance)
Lack of support/fairness → AJC	0.92	0.018	[0.88, 0.96]	0.846
Perceived busyness → AJC	0.84	0.025	[0.79, 0.89]	0.706
Interpersonal strain/conflict → AJC	0.88	0.021	[0.84, 0.92]	0.774
self-bias/perceived prejudice → AJC	0.86	0.023	[0.82, 0.91]	0.740

Note. AJC = adverse job characteristics. R^2 indicates the proportion of variance in each first-order dimension explained by the second-order AJC factor.

Part E: Perceived Organizational Environment – First-Order Loadings

Item Code	Abbreviated Item Content	λ	Robust SE	95% CI	Standardized Residual Variance

Item Code	Abbreviated Item Content	λ	Robust SE	95% CI	Standardized Residual Variance
POE1	The work environment atmosphere is harmonious and pleasant	0.89	0.020	[0.85, 0.93]	0.208
POE2	I have a comfortable and free research atmosphere	0.88	0.021	[0.84, 0.92]	0.226
POE3	I often receive affirmation and support from others	0.87	0.022	[0.83, 0.91]	0.243
POE4	The research environment allows me to gain research inspiration	0.86	0.023	[0.82, 0.91]	0.260
POE5	The institution provides sufficient academic exchange opportunities	0.85	0.024	[0.80, 0.90]	0.278
POE6	The discipline encourages excellent research achievements	0.85	0.024	[0.80, 0.90]	0.278

Note. POE = perceived organizational environment. All loadings are statistically significant at $p < .001$.

Supplementary Table S2. Complete Item Wording, Coding, and Content-Overlap Information

Note. This table provides complete information for all retained and deleted measurement items, including original item numbers, final analysis codes, dimension assignments, English translations, the Chinese wording administered to participants, reverse-scoring status, retention or deletion status, and identified areas of potential content overlap. Original item numbers and final analysis codes are reported separately because the analysis codes were retained to match the final dataset and Mplus syntax. N = 532.

Original Item #	Final Code	Dimension	Item Content (English)	Item Content (Chinese)	Reverse Scored	Status	Potential Overlap
1	WA1	Normlessness	The work assigned to me by the institution often makes me feel confused and at a loss.	单位分配给我的工作经常让我感到困惑和迷茫。	No	Retained	None identified
3	WA3	Normlessness	In my institution, only by maintaining good interpersonal relationships can one get promoted smoothly.	在我所在的单位,只有维持良好的人际关系才能顺利晋升。	No	Retained	None identified
4	WA4	Normlessness	The rewards and punishments in my institution are unclear; working more or less makes no difference.	单位的奖惩不明确,干多干少一个样。	No	Retained	Overlap with AJC10 (effort–reward imbalance)
5	WA5	Normlessness	To get promoted in my institution, one must maintain good relationships with leaders.	在单位要想得到晋升,必须与领导维持良好关系。	No	Retained	None identified
6	WA6	Normlessness	My work is heavy, but I do not receive corresponding rewards.	我的工作很繁重,但得不到相应的回报。	No	Retained	Overlap with AJC10 (effort–reward imbalance)

11	WA11	Normlessness	Promotion in my institution basically depends on relationships rather than ability.	单位的晋升基本靠关系而不是能力。	No	Retained	None identified
7	WA7	Powerlessness	My work is routine and lacks creativity.	我的工作程式化,缺乏创新性。	No	Retained	None identified
8	WA8	Powerlessness	The greatest meaning of my work to me is just this salary.	工作对我最大的意义就是这份工资。	No	Retained	None identified
9	WA9	Powerlessness	To maintain my livelihood, I have to continue with my current work.	为了维持生计,我不得不继续从事目前的工作。	No	Retained	None identified
10	WA10	Powerlessness	I do not like my work; this job cannot realize my value.	我并不喜欢我的工作,这份工作无法实现我的价值。	No	Retained	None identified
12	WA12	Powerlessness	My work can showcase my talents very well.	我的工作能很好地展示我的才能。	Yes	Retained	None identified
2	WA2	Powerlessness	In my work, I often have to do things I do not want to do.	在工作中我经常不得不做自己不愿意做的事。	No	Retained	None identified
13	WA13	Social alienation	I get along very well with my colleagues.	我与同事们相处得很好。	Yes	Retained	None identified

15	—	Social alienation	I can make quite a few close friends at my workplace.	在单位我能结交到不少知心朋友。	Yes	DELETED	Low loading (λ = 0.38); weak CITC (0.24)
14	WA15	Social alienation	Disagreements with colleagues on work issues do not affect our relationships.	工作问题上与同事有分歧不会影响我们的关系。	Yes	Retained	None identified
17	WA17	Social alienation	When problems occur at work, I often cannot get sincere help from colleagues.	工作中出现问题时,我往往得不到同事真诚的帮助。	No	Retained	Overlap with AJC2 (colleague support)
18	WA18	Social alienation	Sometimes I feel like I am alone in the institution.	有时我觉得自己在单位形单影只。	No	Retained	Overlap with AJC18 (organizational belonging)
25	WA25	Social alienation	I often feel lonely at work.	我在工作中经常感到孤独。	No	Retained	Overlap with AJC2, AJC12–14 (interpersonal strain)
19	WA19	Meaninglessness	Regarding promotion in my institution, I think the prospects are dim.	对于在单位的晋升,我觉得前景渺茫。	No	Retained	Overlap with AJC4 (lack of support in promotion)
20	WA20	Meaninglessness	Current busy work lays the foundation for doing higher-level things in the future.	目前繁忙的工作是为将来做更高层次的事打基础。	Yes	Retained	None identified

21	—	Meaninglessness	Overall, I feel that my current work is still meaningful.	总体来看,我觉得目前的工作还是有意义的。	Yes	DELETED	Low loading (λ = 0.41); weak CITC (0.28)
22	WA22	Meaninglessness	Overcoming difficulties at work is one of the great pleasures in my life.	克服工作中的困难是我人生一大乐趣。	Yes	Retained	None identified
23	WA23	Self-estrangement	I always feel like I am a failure.	我总觉得自己是个失败者。	No	Retained	Overlap with AJC15–16 (self-perceived ability)
24	WA24	Self-estrangement	I often doubt my work ability.	我经常怀疑自己的工作能力。	No	Retained	Overlap with AJC15–16 (self-perceived ability)
26	WA26	Self-estrangement	No matter how hard I try, I cannot get others' recognition.	无论我如何努力,都得不到他人的认可。	No	Retained	Overlap with AJC1, AJC4 (lack of recognition/support)
16	WA16	Self-estrangement	I am just an ordinary member; as long as I do not make mistakes, that is good enough.	我只是普通一员,只要不出错就好。	No	Retained	None identified

Part A: Work Alienation Scale (24 Retained Items + 2 Deleted Items)

Item #	Final Code	Dimension	Item Content (English)	Item Content (Chinese)	Reverse Scored	Potential Overlap
1	AJC1	Lack of support/fairness	I do not feel support from leaders.	我感受不到来自领导的支持。	No	Overlap with POE3 (support); WA26 (recognition)
2	AJC2	Lack of support/fairness	I do not feel support from colleagues.	我感受不到来自同事的支持。	No	Overlap with WA17, WA25 (colleague support/isolation)

3	AJC3	Lack of support/fairness	In my work, I encounter "exploitation" from colleagues or leaders.	在工作中我遇到同事或领导的"剥削"。	No	None identified
4	AJC4	Lack of support/fairness	I do not feel support in academic publication and project application.	在学术发表和项目申请方面我得不到支持。	No	Overlap with WA19 (promotion prospects); POE5 (academic exchange)
5	AJC5	Lack of support/fairness	I often cannot find research collaborators.	我经常找不到科研合作者。	No	None identified
6	AJC6	Lack of support/fairness	Some leaders create obstacles for my work.	有些领导给我的工作制造障碍。	No	None identified
7	AJC7	Perceived busyness	This job leaves me almost no personal time.	这份工作让我几乎没有个人时间。	No	None identified
8	AJC8	Perceived busyness	I feel busy almost every day.	我几乎每天都感到很忙。	No	None identified
9	AJC9	Perceived busyness	Since starting this job, I hardly know when I am truly "off work."	自从做这份工作,我几乎不知道什么时候是真正的"下班"。	No	None identified
10	AJC10	Perceived busyness	In terms of income, my efforts far exceed what I get.	就收入而言,我的付出远远超过我得到的。	No	Overlap with WA6, WA4 (effort–reward imbalance)
11	AJC11	Perceived busyness	What I have to do each day is always more than I imagined.	每天要做的事总是超过我想象的。	No	None identified
12	AJC12	Interpersonal strain/conflict	Dealing with interpersonal relationships often makes me feel tired and annoyed.	处理人际关系经常让我感到疲惫和烦恼。	No	Overlap with WA25 (loneliness); WA17 (colleague difficulties)
13	AJC13	Interpersonal strain/conflict	Interpersonal matters often affect my work mood.	人际方面的事经常影响我的工作心情。	No	Overlap with WA social alienation items
14	AJC14	Interpersonal strain/conflict	I hope there is less internal friction from interpersonal relationships.	我希望人际关系方面的内耗少一些。	No	Overlap with WA social alienation items
15	AJC15	self-bias/perceived prejudice	I think I am not diligent enough.	我觉得自己不够勤奋。	No	Overlap with WA23–24 (self-perceived failure/ability)

16	AJC16	self-bias/perceived prejudice	I think my ability is insufficient.	我觉得自己能力不足。	No	Overlap with WA23–24 (self-perceived failure/ability)
17	AJC17	self-bias/perceived prejudice	I think the institution could replace me at any time.	我觉得单位随时可以替换掉我。	No	None identified
18	AJC18	self-bias/perceived prejudice	I think I am not a real member of the institution.	我觉得自己不是单位真正的一员。	No	Overlap with WA18 (organizational belonging/isolation)

Part B: Adverse Job Characteristics Scale (18 Items)

Part C: Perceived Organizational Environment Scale (6 Items)

Item #	Final Code	Item Content (English)	Item Content (Chinese)	Reverse Scored	Potential Overlap
1	POE1	The work environment atmosphere is harmonious and pleasant.	工作环境氛围和谐愉快。	No	General positive climate
2	POE2	I have a comfortable and free research atmosphere.	我拥有舒适自由的科研氛围。	No	General organizational climate; no close item-level counterpart identified
3	POE3	In my work environment, I often receive affirmation and support from others.	在工作环境中我经常得到他人的肯定和支持。	No	Overlap with AJC1–2 (lack of support from leaders/colleagues)
4	POE4	The research environment allows me to gain research inspiration.	科研环境能让我获得研究灵感。	No	None identified
5	POE5	The institution provides sufficient academic exchange opportunities.	单位提供充足的学术交流机会。	No	Overlap with AJC4–5 (lack of academic support/collaborators)
6	POE6	The discipline encourages excellent research achievements and provides corresponding rewards.	学科鼓励优秀科研成果并给予相应奖励。	No	Overlap with WA4, WA6, AJC10 (reward system)

Supplementary Table S3. Exploratory Sensitivity Analyses of Conceptually Overlapping Content

Analysis	WA-AJC r	HTMT [95% CI]	AVE WA	√AVE WA	AVE AJC	√AVE AJC	Fornell-Larcker conclusion	B	CR2 SE	β	CR2 df	CR2 p	Wild-bootstrap p
Primary measures	.847	.886 [.861, .910]	0.748	0.865	0.767	0.876	Satisfied	.741	.030	.745	6.9	<.001	.002
Excluding SBP dimension	.782	.820 [.793, .849]	0.757	0.870	0.702	0.838	Satisfied	.698	.036	.682	6.4	<.001	.002
Excluding six overlapping items	.761	.798 [.769, .828]	0.762	0.873	0.707	0.841	Satisfied	.671	.037	.658	6.3	<.001	.003

Note. N = 532 teachers nested within eight universities. WA = work alienation; AJC = adverse job characteristics; SBP = self-bias/perceived prejudice; AVE = average variance extracted. For the primary higher-order WA and AJC constructs, AVE was calculated from the standardized second-order loadings of the corresponding first-order dimensions. In the sensitivity rows, AVE values are the squared equivalents of the reported √AVE values and are rounded to three decimals. The Fornell-Larcker criterion was evaluated by comparing both construct-specific √AVE values with the absolute WA-AJC correlation. The analyses were exploratory and post hoc. The first analysis removed the SBP dimension; the resulting three-dimension second-order AJC specification was minimally identified. The second analysis removed AJC Items 2, 15, 16, and 18 and WA Items 18 and 24; because both measures changed, its regression coefficient is not directly comparable with the primary coefficient. All three regressions used the same moderation-model specification, with AJC and POE mean-centered and the AJC × POE term retained solely for model comparability. B is the conditional AJC association when POE is at its sample mean. Regression significance was not interpreted as evidence of discriminant validity. All estimates came from separately re-estimated models.

Supplementary Table S4. Complete Hierarchical Regression Results: Predicting Work Alienation

Part A: Step 1 - Background Variables Only

Variable	B	SE	β	95% CI (β)	p	VIF
Gender (0=male, 1=female)	-0.061	0.038	-0.064	[-0.140,0.012]	.142	1.08
Age (continuous, years)	0.014	0.012	0.049	[-0.039, 0.137]	.258	1.24
Marital status (0=unmarried, 1=married)	-0.132	0.045	-0.139	[-0.230,-0.048]	.004	1.15
Education (0=non-doctoral, 1=doctoral)	-0.045	0.048	-0.047	[-0.144, 0.050]	.281	1.12
Rank (0=junior, 1=intermediate+)	0.038	0.042	0.040	[-0.044,0.124]	.359	1.28
Discipline (0=humanities, 1=STEM)	0.066	0.042	0.069	[-0.016, 0.154]	.118	1.09
Admin role (0=no, 1=yes)	-0.058	0.041	-0.061	[-0.144, 0.022]	.162	1.11
Institution (0=non-DFC, 1=DFC)	0.121	0.044	0.127	[0.037, 0.217]	.006	1.19
Income (continuous, ¥10,000)	-0.024	0.010	-0.108	[-0.198,-0.018]	.018	1.16

Model Summary: $R^2 = .061$, Adjusted $R^2 = .045$, $F(9, 522) = 3.77$, $p < .001$

Part B: Step 2 - Background Variables + Adverse Job Characteristics Dimensions

Variable	B	SE	β	95% CI (β)	p	VIF
Gender (0=male, 1=female)	-0.012	0.018	-0.013	[-0.047,0.021]	.505	1.12
Age (continuous, years)	-0.003	0.006	-0.011	[-0.056, 0.034]	.624	1.28
Marital status (0=unmarried, 1=married)	-0.028	0.021	-0.029	[-0.070,0.012]	.186	1.18
Education (0=non-doctoral, 1=doctoral)	-0.015	0.022	-0.016	[-0.059, 0.027]	.498	1.14
Rank (0=junior, 1=intermediate+)	0.008	0.019	0.008	[-0.030,0.046]	.682	1.32
Discipline (0=humanities, 1=STEM)	0.018	0.019	0.019	[-0.020,0.058]	.351	1.12
Admin role (0=no, 1=yes)	-0.011	0.019	-0.012	[-0.049,0.025]	.548	1.14

Institution (0=non-DFC, 1=DFC)	0.024	0.020	0.025	[-0.015,0.065]	.235	1.22
Income (continuous, ¥10,000)	-0.006	0.005	-0.027	[-0.064,0.010]	.216	1.18
Lack of support and fairness	0.601	0.034	0.619	[0.552, 0.686]	<.001	2.68
Perceived busyness	0.115	0.028	0.122	[0.060, 0.184]	<.001	1.94
Interpersonal strain and conflict	0.079	0.029	0.084	[0.027,0.141]	.007	2.12
Self-bias/perceived prejudice	0.150	0.027	0.158	[0.103, 0.213]	<.001	2.45

Model Summary: $R^2 = .767$, Adjusted $R^2 = .761$, $F(13, 518) = 131.17$, $p < .001$

Change Statistics: $\Delta R^2 = .706$, $\Delta F(4, 518) = 392.39$, $p < .001$, Cohen's $f^2 = 3.03$

Note. N = 532. B = unstandardized coefficient; SE = standard error; β = standardized coefficient; CI = confidence interval; VIF = variance inflation factor; DFC = Double First-Class university; STEM = science, technology, engineering, and mathematics. All standard errors and tests are based on ordinary regression; cluster-adjusted results are reported in Table 7. The source dimension labelled "self-bias/perceived prejudice" is retained in tables for transparency and is operationally interpreted as perceived diminished professional standing, combining self-devaluation and perceived organizational marginalization.
